# Dr. Mossman, on Apoplexy

**Published:** 1802-03

**Authors:** Geo. Mossman

**Affiliations:** Bradford


### *To the Editors of the Medical and Phyſical Journal.*

GENTLEMEN,

IN your 34th Number, is recorded a profeſſional Controverſy on the ſubject of *Apoplexy*. As a prelude to the projected diſcuſſion of this diſeaſe, it may be admiſſible to lament the want of candour which is ſo frequently manifeſted in Medical Controverſies. Illiberality is the bane of ſcience, the diſgrace of individuals, and a deformity too frequently attached to the profeſſors of our art.

From the firſt bluſh of the narrative before me, it is impoſſible not to cenſure the conduct of the *Surgeon*. I chooſe to give full credit to his own repreſentation, and upon that alone I ground my verdict. His reaſons for *not consulting with Dr. Langslow* are veiled in impenetrable myſtery; but, whatever they may be, I am perſuaded, that a diſcloſure cannot do honor to to his judgement or to his feelings. It is acknowledged that a fellow creature, labouring under a moft dangerous malady, was denied the benefit of a *consultation*; and till I am better inftructed, I muft believe, that a valuable lady would have perifhed, had her life depended upon a friendly profeffional intercourfe between *Dr. Langslow*, and *one of her attendant surgeons*.

*Mr. Primrose*'s conduct was, apparently, unexceptionable.

On the face of the narrative, there is nothing ungentlemanlike, or inconfiftent with profeffional etiquette, on the part of *Dr. Langslow*. So much for the Controverfy.

I fhall now attempt an Inquiry into the nature of *Apoplexy*; and I fincerely hope, that, through the medium of your excellent Journal, the difeafe will undergo a thorough examination. I flatter myfelf too, that, in the Inquiry to be inftituted, the caufe of fcience alone will be the object of difpute. If the chief duty of our art be an alleviation of the ills connected with our nature; and if that end be atchieved, it is not benevolent to be jealous of the means.

Much has been written about the nature and cure of *Apoplexy*; and a man who is wedded to no fyftem, finds it difficult to form his opinion; becaufe, happily for our fpecies, this difeafe does not occur fo frequently, as to furnifh any individual practitioner with much actual obfervation. That he may fettle his faith, he muft recur to the records of others. He muft collate, arrange, and compare the hiftories fo detailed. Above all, he muft advert with particular attention to appearances after death. From inquiries fo conducted, I am perfuaded that inferences may be eftablifhed, approximating to the truth, nearly enough for fuccefsful practice.

There is, in authors a very ftriking coincidence of defcription of the *living* and *dead phænomena* of *Apoplexy*. The extreme points being thus fettled, it is to be expected that the intermediate fteps fhould be traced with the moft perfect agreement. That the curative indications of *apoplexy*, however, are, in different hands, repugnant to each other, is fufficiently evident from the prefent Controverfy. The fchool divifion of this difeafe into *sanguineous* and *serous*, is now, I believe, very generally, and I think juftly, exploded; for were the diftinction demonftrated to be true, it does not admit of a beneficial application in practice. I fhall therefore difmifs this diftinction without a comment, and confider *Apoplexy* as arifing from *something* COMPRESSING *the brain, and thereby destroying the energy of that organ*. This definition, I imagine, will embrace almoft every form of the difeafe. I do not affect to mark with precifion the degrees of diforganization attached to thofe cafes of *Apoplexy* which terminate in recovery; but in fatal cafes, where an infpection

ſpection has been permitted, it is certainly true, that a *turgescency* of the vaſcular ſyſtem, or an *effusion* of fluid matter, have been conſtantly demonſtrated.

If thoſe who have recovered from, and thoſe who have periſhed by, *Apoplexy*, have exhibited features of diſeaſe preciſely ſimilar, I think it is probable that the favorable and the fatal caſes have had their origin in the ſame cauſe, more or leſs powerfully applied. A brainular plethora is rendered probable in favorable caſes of the diſeaſe, by its being demonſtrated in caſes which have terminated fatally; but whether this *turgescency* be excluſively attached to the *arterial system*, or to the *system of recurrent vessels*; or whether *effusion* (when it occurs) be *arterial* or *venous*, are queſtions which will, for ever, perhaps, demand a doubt; nor do I think it would be of high practical intereſt to put the matter beyond ſuſpicion. The multiplied evidence of Diſſection will prove, that in the ſame patient the veſſels of the head have been found, ſome in a *turgid*, others in a *ruptured* ſtate. If this be true, it ought not merely to influence but to fix the indication of cure; for whether *turgescency* be preſent without *effusion*, or there be a combination of both, I am perſuaded that the ſame curative inſtruments ought to be employed.

In the treatment of *every Case of Apoplexy*, I preſume upon the preſence of COMPRESSION; for altho' I admit that a ſlow and inſidious diſeaſe may aſſail the brain itſelf, and may eventually induce *Apoplexy*, yet, as ſuch morbid phænomena are placed beyond the reach of our diagnoſtic art, a perfect knowledge of their exiſtence cannot *practically* avail us.

I ſhall never be able ſatisfactorily to prove it, but I am ſtrongly inclined to believe, that, in all caſes of convaleſcent *Apoplexy*, there is no material *rupture*, but merely a *turgescency* of the *vascular system*. I ground this belief upon a conviction that a *sudden* effuſion of any *large quantity* of fluid upon the brain, is incompatible with animal life.*

It muſt be admitted, that patients having a large collection of fluid in the ventricles of the brain, have lived for a conſiderable time; but it ſhould be obſerved, that that fluid has been ſlowly effuſed or ſecreted, and the parts gradually adapted to its preſence. *Hydrocephalus* is, I believe, never cured; for were it poſſible to remove the water by a wiſh, I imagine that the collapſe would be fatal to the patient: It is ſcarcely poſſible, I think, under circumſtances of *confirmed Hydrocephalus*, for the moſt ſanguine practitioner to expect a gradual abſorption.

I do

* Slight effuſions have often occurred without a fatal event. See Doctor Baillie, &c.

I do not pretend to trace the *arterial system* of the brain to its minuteſt ramifications, or to mark with preciſion the point at which they become returning veins; but, from the collated evidence before me, I am inclined to believe in *arterial* not *venous effuſion.* The enunciation of this opinion, however, cannot affect the indication of cure; and I would induce a diminution of brainular plethora by *local and general bleedings*, *bliſters*, *and purgative clyſters.*

The exhibition of *Emetics*, is, in my mind, a practice highly dangerous; nothing, ſurely, can be leſs indicated. The affection of the ſtomach, which firſt ſuggeſted their employment, is very certainly a *ſymptomatic* diſeaſe only; for in all injuries done to the brain, of whatever nature they may be, there is uniformly an affection of the ſtomach: But, with the exception of injury ariſing from *Apoplexy*, I do not know that an *Emetic* was ever thought of to cure any *idiopathic* brainular diſeaſe.

Original thinking is a rare quality; and if this be admitted, I can explain why a great man ſhould be copied, even in his *errors:* But it is not ſo eaſy to ſay, why the expanded mind of a *Fothergill* ſhould miſtake an effect for its cauſe. The ſanction, however, of that illuſtrious obſerver, has long ſince ceased to operate upon the practice of men of ſcience; for a moment's reflection muſt convince every one, that, were the doctrine of the ſtomach's mechanical preſſure true, no diſeaſe would be ſo common as *Apoplexy*; and no diſeaſe would be ſo eaſily, and ſo immediately, remedied. There needs no ſpecification of the means. Every man who has treated *Apoplexy*, muſt have witneſſed inſtant relief by the operation of *bleeding*; would this have happened, had the diſeaſe been conſtituted by *diſtenſion of the ſtomach?* After a *full meal*, it is true, that *Apoplexy* very frequently occurs; but this proves nothing more than that the ſtimulus of the food, by increaſing arterial impetus, operates as an exciting cauſe of the diſeaſe.

If the phænomena of *Apoplexy* have their origin in *turgescency*, nothing is ſo likely as *vomiting* to produce *rupture* and *effuſion*; and if thoſe ſymptoms be preſent, nothing is ſo likely as *vomiting* to increaſe both. In fine, I can ſcarcely ſuppoſe any practice fraught with greater danger than the adminiſtration of *Emetics* during the attack of an *Apoplexy*; and their employment has been reprobated by the higheſt profeſſional authorities; I cannot quote greater than the juſtly celebrated *Morgagni*, and the enlightened *Cullen.*

I am, &c.

*Bradford*, 15 *Feb.* 1802. GEO. MOSSMAN.

